# Effect of Type of Pregnancy on Transcriptional and Plasma Metabolic Response in Sheep and Its Further Effect on Progeny Lambs

**DOI:** 10.3390/ani10122290

**Published:** 2020-12-03

**Authors:** María Gallardo Paffetti, Juan Cárcamo, Luis Arias-Darraz, Carlos Alvear, Javier Ojeda

**Affiliations:** 1 Escuela de Medicina Veterinaria, Facultad de Ciencias, Universidad Mayor, Santiago PO Box 8580745, Chile; maria.gallardo@umayor.cl (M.G.P.); calvears@yahoo.com (C.A.); 2 Centro FONDAP, Interdisciplinary Center for Aquaculture Research (INCAR), Facultad de Ciencias, Universidad Austral de Chile, Valdivia PO Box 567, Chile; l.arias.darraz@gmail.com; 3 Instituto de Ciencias Clínicas Veterinarias, Facultad de Ciencias Veterinarias, Universidad Austral de Chile, Valdivia PO Box 567, Chile; jojeda@uach.cl

**Keywords:** angiogenesis, lactogenesis, metabolic indicators, lamb muscle

## Abstract

**Simple Summary:**

The present study was carried out in order to determine the effect of type of pregnancy on the mammary gland development, evaluated through the transcriptional expression of genes that are associated to angiogenesis and cell turnover/lactogenesis and the metabolic response of the animals. For this, six twin and seven single-bearing ewes were fed with naturalized pasture from day −45 pre-partum until day +70 post-partum, taking samples of mammary tissue and plasma at different times from the birth until weaning. The results showed the type of pregnancy could only explain a few differences in the transcriptional expression of in some genes that are involved in angiogenesis and cell turnover/lactogenesis in the mammary gland tissue, which had no impact on the metabolic status of ewes or the metabolic response in plasma, performance, and muscle transcriptional expression of the lambs.

**Abstract:**

The following study was performed in order to determine the effect of type of pregnancy on the transcriptional expression of genes that are engaged in angiogenesis and cell turnover/lactogenesis in the ewe mammary gland, evaluating its impact on the plasma metabolic response. In addition, an assessment of its further influence on plasma metabolic response, performance, and muscle transcriptional expression of lipogenic enzymes in progeny lambs was made. Thirteen Ile de France sheep (six twin- and seven single-bearing ewes) were allocated to graze ad libitum naturalized pasture from d 45 pre-partum to day 70 post-partum, while keeping their lambs on the same diet until day 60 after weaning. The samples were collected at different times and analyzed by qRT-PCR and plasma metabolic indicators. The data were processed using SPSS package. The results showed that twin-bearing ewes overexpressed VEGFR1 at birth, and BCL2 at birth and day 35 post-partum; however, single-bearing ewes overexpressed CAIV and IGF1 at day 35 post-partum. Similar metabolite concentrations in blood plasma were found between groups of ewes. The plasma metabolic response in lambs was similar between groups and it did not influence their performance, where a similar transcriptional expression of lipogenic enzymes in muscle was observed. Therefore, the type of pregnancy can explain the slight differences in mRNA expression that were found in angiogenesis and cell turnover/lactogenesis in mammary gland, although these differences not only did not affect the plasma metabolic response in ewes, but they also had no influence on plasma metabolic response, performance, and muscle transcriptional expression of their lambs.

## 1. Introduction

Pasture-based sheep production raising has serious problems that are related to lamb survival and growth until weaning [1], especially in twin-bearing ewes [2], where increasing energy demands have been observed when compared to ewes carrying a single fetus [3].

Some studies have reported a direct association between sheep prolificacy and milk yield [4]. At this point, there are precedents indicating that angiogenesis and cell proliferation are processes that are outdated in time [5]. During early lactation, the mammary parenchyma regulates its own vascularization [6], and certain proteins play a role as angiogenic factors, such as CAIV and VEGF, and their receptors VEGFR1 and VEGFR2, ANGPT1 and ANGPT2 (an antagonist of ANGPT1 and inducer of endothelial cell apoptosis), and RTK (with high-affinity cell surface receptors for many polypeptides growth factors, cytokines, and hormones) [7,8]. Furthermore, some other proteins that are associated to cell turnover/lactogenesis are LALBA, related to the secretion of milk [9], BAX, BCL2, CCND1, IGF1, and its receptor IGF1R, plus IGFBP3 and IGFBP5, TGFB1 and its receptors TGFB1R1 and TGFB1R2, and ultimately LPTR [10].

It is known that the milk production and its persistence are influenced by the metabolic status of the animals [11], highlighting plasma albumin as a marker of the nitrogen metabolism [12], cholesterol as an indicator of the lipid metabolism [13], total protein as an indicator of protein synthesis level [14], and finally plasma urea, as an indicator of the biological response to protein or energy intake [15,16]. Although glucose is important to maintain vital organs, together with fetal growth, also having a role in lactation process [17], it is not a good indicator of energy status in ruminants [18], with β-hydroxybutyrate (BHB) being a better energy parameter [19].

Taking into account the highest percentage of stearic acid (18:0), the meat from sheep has, when compared to other types of meat and knowing that there is a relationship between the stearic acid content and the toughness of the meat [20], so any factor that increases the conversion from 18:0 to 18:1 cis9 will increase the tenderness of the meat.

Thus, the 18:1 cis9 proportions that are present in muscle phospholipids from young animals would influence the muscle fatty acid composition; however, as body fat increases, neutral lipids would predominate over muscle phospholipids [21]. The long chain ω3-polyunsaturated fatty acids (PUFA) are mainly incorporated within the membrane phospholipids, instead of being incorporated within the triacylglycerols, thus allowing for the intramuscular composition of fatty acids of the meat to be manipulated, without greatly increasing fatness [22].

It has been reported that pasture-based diets, which are high in ω3-polyunsaturated fatty acids (PUFA), can inhibit the mRNA expression of some genes that are involved in lipid biosynthesis, such as Acetyl-CoA carboxylase (ACC), Fatty acid synthase (FAS), Stearoyl CoA desaturase 1 (SCD1), Sterol regulatory element binding transcription factor 1c (SREBP1c), Lipoprotein lipase (LPL), and Peroxisome proliferator-activated receptor gamma (PPARγ) [23,24,25], decreasing the de novo synthesis of fatty acids and tissue-specific responses in ruminants [26,27,28].

Therefore, the present study of molecular expression of lipid metabolism emerges as one of the objectives to consider in lamb meat research. Thus, not only the diet, but also the influence of the ewes on the lamb meat, are factors that must be evaluated. When considering the given information, the formulated hypothesis is the type of pregnancy (single or twin) influences the transcriptional expression of genes that are associated to angiogenesis and cell turnover/lactogenesis in the mammary gland tissue, having a further impact on the plasma metabolic response in the ewes and their lambs, finally affecting the performance and transcriptional expression of genes that are involved in the lipid metabolism of the lamb muscle. The first objective was to determine the effect of the type of pregnancy on the transcriptional expression of genes that are involved in angiogenesis and cell turnover/lactogenesis in the mammary gland of twin- (TP) and single-bearing ewes (SP) fed naturalized pasture (NP), and its further effect on the plasma metabolic response on them. The second objective determined the effect of the type of pregnancy on the plasma metabolic response, performance, and muscle transcriptional expression of lipogenic enzymes in progeny lambs.

## 2. Materials and Methods

### 2.1. Bioethics

The Committee for the Ethical Use of Animals in Experiments of the Universidad Austral de Chile (Valdivia, XIV Region, Chile) approved the methodology used in this study (N°241/2015).

### 2.2. Location

The experiment was conducted in a farm located 12 km southeast of Villarrica, IX Region, Chile (3916′0″ S,7213′0″ E), from July 2017 to February 2018.

### 2.3. Animals and Sampling

A sample group of thirteen Ile de France, third-birth sheep formed by six twin- and seven single-bearing ewes, with a similar body condition score (BCS, 3.0), were randomly selected from a large, free-grazing flock feeding on NP, a successional pasture post cultivation, dominated by species, such as *Agrostis tenuis*, *Holcus lanatus*, and *Trifolium repens* [29]. Although the sample number of animals for each physiological stage is not very high, this number is similar to other publications in the area, allowing for determining significant differences between treatments [10,25,30]. One week before birth, the ewes were held in a pen for three weeks, a period during which they were fed naturalized pasture hay (NPH), supplemented with lupine (88.90% dry matter (DM), 17.17% crude protein (CP), 3.08 Mcal metabolizable energy (ME) (kg DM^−1^), 52.72% neutral detergent fiber (NDF), and 3.37% total ashes (TA)) in order to meet their requirements and then returned to graze NP. The ewes were monitored from d 45 pre-partum to day 70 post-partum, at a time in which they left the study. On the other hand, their lambs were monitored from the moment of lambing, being later weaned and kept under the same pasture until the end of the experiment on day 60 after weaning. The samples of the mammary gland were obtained at day 0 (time 1), day 35 (time 2), and day 70 (time 3) post-partum and analyzed while using the Delta-Delta-Ct (ddCt) method for qRT-PCR data. The procedure that was employed in the biopsy sampling of the mammary gland followed the protocol described by Nielsen [31]. Thus, plasma samples from ewes were collected at day 30, and milk samples were retrieved at days 0, 35, and 70 post-partum. In lambs, performance jointly with plasma metabolic response measurements were made at birth, day 35, and day 70 after birth (weaning), and also at day 30 and 60 after weaning in order to determine albumin, cholesterol, total protein, urea, and β-hydroxybutyrate (BHB) levels. The blood sampling procedure was performed according to the protocol that was described by Gallardo et al. [32]. The samples were stored at −80 °C for later analyses at the Institute of Biochemistry and Microbiology, belonging to the Universidad Austral de Chile (UACh). The biopsy samples from *Longissimus dorsi* muscle were taken at weaning, day 30 and day 60 after weaning, according to the protocol that was described by Gallardo et al. [29]. Later, these samples were kept in RNA Safer Stabilizer Reagent (E.Z.N.A.) at −80 °C for RNA extraction in the laboratory facilities at the Institute of Biochemistry and Microbiology, belonging to the Universidad Austral de Chile.

At the beginning of each experimental period, the chemical composition of NPH and NP was assessed from three composite samples each, which were obtained from 10 randomly chosen bales, and from three 1-ha area paddocks, respectively. The chemical analyses were performed at the Animal Production Institute of the Universidad Austral de Chile in order to determine dry matter (DM) content, crude fat (CF) [33], crude protein (CP) [34], neutral detergent fiber (NDF) [35], and metabolizable energy (ME). Metabolizable energy estimations were made with a regression analysis while using a “D” value (digestible organic matter/DM × 100) assessed in vitro [36], according to Goering and Van Soest [37].

### 2.4. qRT-PCR Analysis

The qRT-PCR was performed through a Lightcycler Mx3005P (Agilent Technologies, Santa Clara, CA, USA). The RNA extraction (from 50 mg of mammary gland and muscle samples) was carried out while using TRIzol™ Reagent (ThermoFisher, Waltham, MA, USA), according to the protocol that was indicated by the manufacturer. The RNA elution was carried out using nuclease-free water. The RNA was spectrophotometrically quantified; in addition, the 260/280 and 260/230 ratios were reviewed. The cDNAs were obtained by reverse transcription while using 2 µL of RNA in each case. The reverse transcription was obtained using the M-MLV Reverse Transcriptase (ThermoFisher, Waltham, MA, USA). The qRT-PCR were made by using 5-μL reaction mixes of Brilliant II SYBR® Green Master Mix (Agilent Technologies, CA, USA), 0.5 μL forward/reverse primer solution (0.2 μmol/L) (designed with Primer-BLAST and further analyzed by AmplifX), and 1 μL cDNA template (1:5 dilution ratio) to a thermo cycling program for 10 s at 95 °C, 30 s at 60 °C, and 45 s at 70 °C (45 cycles). Specific oligonucleotides for mammary gland transcripts were designed (Table 1). Because β-actin transcript was stably expressed, it was used as a reference gene for gene expression normalization. The oligonucleotides that were designed to evaluate lipid metabolism, such as ACCA (NM_001009256.1), FASN (XM_004013447.1), SCD1 (NM_001009254.1), SREBP1c (XM_004013336.1), LPL (NM_001009394.1), PPARγ (NM_001100921.1), and actin (NM_001009784.1), are detailed in Gallardo et al. [25]. The relative mRNA expression was determined through the comparative efficiency-corrected ΔΔCT method [38]. The significance of results were determined according the *p*-values that were obtained for all comparisons.

### 2.5. Animal Performance

The measurements on lambs included determinations of initial and final live body weight (LBW; kg) with a Diamond Series A-04 digital scale, performed from lambing until day 60 after weaning during the whole experimental period, and evaluations of average daily gain (ADG; kg) at day 35 after birth, weaning, day 30 post-weaning, and day 60 post-weaning.

### 2.6. Metabolic Determining

The metabolic response (during fasting) was determined at day 30 pre-partum (time 0) in ewes, and at birth (time 1), day 35 post-partum (time 2), and day 70 post-partum (time 3) in ewes and their lambs. For that, the blood samples (5 mL) that were obtained by jugular venipuncture early in the morning were collected in heparinized tubes, being centrifuged at 2500× *g* for 4 min and the plasma was collected and stored at −20 °C for further analysis. The plasma albumin (BCG, WienerLab^®^, Rosario, Argentina, no 1690008), total cholesterol (CHOD-PAP, WienerLab^®^, Rosario, Argentina, no 1220114), urea (urease, WienerLab^®^, Rosario, Argentina, no 1810324), total protein (Biuret, WienerLab^®^, Rosario, Argentina, no 1690009), and BHB (Rambut, Randox^®^, Crumlin, County Antrim, UK), also in milk, were determined while using an autoanalyzer (CM250^®^, WienerLab^®^, Rosario, Argentina) and Standatrol SE device (WienerLab^®^, Rosario, Argentina, Lot. no 1804251660) as internal quality control; it is important to mention that all of these procedures were performed at the Clinical Pathology Laboratory belonging to the Institute of Biochemistry and Microbiology of the Universidad Austral de Chile.

### 2.7. Statistical Analysis

The statistical design of the present study was completely randomized. Thus, the effect of time on the chemical composition of pastures was determined by repeated-measures ANOVA, because we used the same animals at different times, analyzed by the GLM procedure while using SPSS Statistics 23.0 for Windows® (IBM Corp, Armonk, NY, USA). Relative gene expression was obtained while using the comparative efficiency-corrected ΔΔCT method and the *p*-values were estimated using the fold change data. Data that were obtained from the plasma metabolic response were analyzed by *t*-test, to assess significant differences between means at *p*-value ≤ 0.05. The statistical model used in this study was: Yij = μ + Pi + eij, where Yij = observation ij; μ = the overall mean; Pi = the fixed effect of type of pregnancy, and eij = random error. The effect of groups and time was examined by factorial ANOVA while using SPSS Statistics 23.0 for Windows^®^ (IBM Corp, Armonk, NY, USA) and Bonferroni test (*p* < 0.05).

## 3. Results

From chemical analyses of pastures, it was possible to determine higher values in DM, TA, CF, and NDF from birth to the end of the trial, as opposed to that seen in CP and ME proportions, which decreased as the experiment advanced (*p* < 0.0001) (Table 2).

### 3.1. Relative mRNA Expression

**Figure 1:**
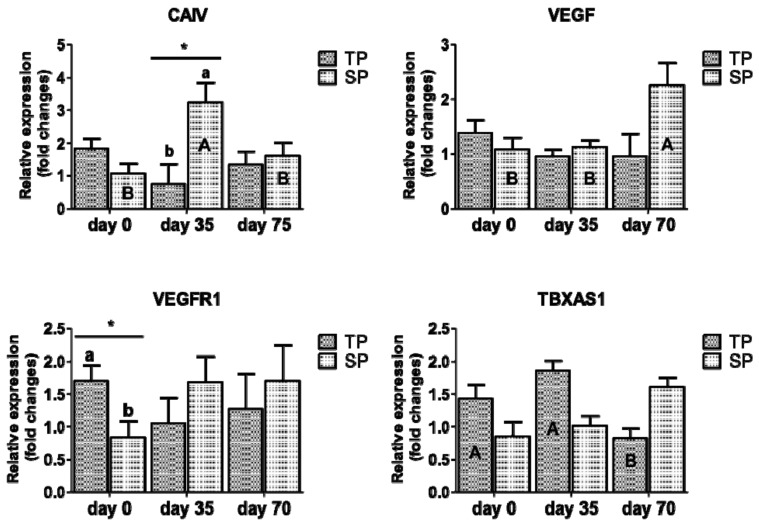
Relative expression of CAIV, VEGF, VEGFR1 and TBXAS1 associated to angiogenesis in the mammary gland of twin- and single bearing ewes according to time (LSM ± SEM). TP: twin-bearing ewes (n = 6); SP: single-bearing ewes (n = 7); CAIV: Carbonic Anhydrase IV; VEGF: vascular endothelial growth factor A; VEGFR1 (FTL1): fms-related tyrosine kinase 1; TBXAS1: thromboxane A synthase. Day 0: birth, day 35: day 35 post-partum and day 70: day 70 post-partum; different small letters (a, b) denote significant differences between groups within each measuring time at *p* ≤ 0.05 (*); ^4^ Different capital letters (A, B) indicate significant differences of each group according time *p* ≤ 0.05 (*).

The relative expression of genes that are associated to angiogenesis (CAIV, VEGF, VEGFR1 and TBXAS1) in the mammary gland of twin- and single bearing ewes according to time (LSM ± SEM) is shown in Figure 1. At day 0, group TP overexpressed VEGFR1 when compared to group SP (*p* < 0.05). At day 35 post-partum, group SP overexpressed CAIV (*p* < 0.05) when compared to group TP. The group SP showed a trend to overexpress ANGPT1 at day 35 and VEGF at day 70, when compared to group TP (*p* = 0.06; data included in Table S3). Regarding time, although TBXAS1 decreased from day 0 to day 70 in group TP (*p* < 0.05), VEGF increased its expression from day 0 to day 70 in the group SP (*p* < 0.05).

**Figure 2:**
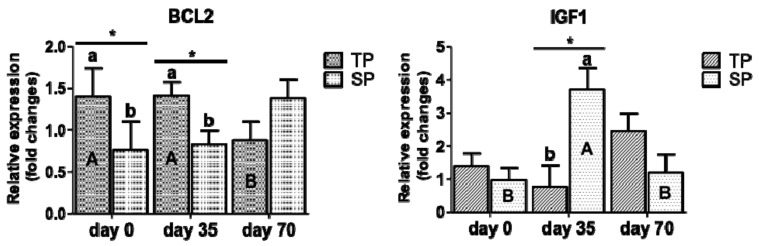
Relative expression of genes associated to cell turnover/lactogenesis in the mammary gland (BCL2 and IGF1) of twin- and single bearing ewes according to time (LSM ± SEM). TP: twin-bearing ewes (n = 6); SP: single-bearing ewes (n = 7); BCL2: B-cell CLL/lymphoma 2; IGF1: insulin like growth factor 1; day 0: birth, day 35: day 35 post-partum and day 70: day 70 post-partum; different small letters (a,b) denote significant differences between groups within each measuring time at *p* ≤ 0.05 (*); Different capital letters (A, B) denote significant differences of each group according to time *p* ≤ 0 (*).

The transcriptional expression that is related to cell turnover/lactogenesis in the mammary gland (BCL2 and IGF1) of twin- and single-bearing ewes according to time (LSM ± SEM) is shown in Figure 2. At day 0 and day 35, the group TP presented a higher expression of BCL2 than the group SP (*p* < 0.05). At day 35 the group SP overexpressed IGF1 (*p* < 0.05) in contrast to group TP. The group TP presented a trend to increase the expression of IGFBP3 at day 0 (*p* = 0.06), but no differences between groups were observed at day 70 (data included in the Table S4). Regarding time, despite that the group TP overexpressed BCL2 at day 0 and day 35 and decreased at day 70 (*p* < 0.05), the group SP overexpressed IGF1 at day 35 and showed a decrease at day 70 (*p* <0.05).

### 3.2. Effect on Metabolic Response

**Figure 3:**
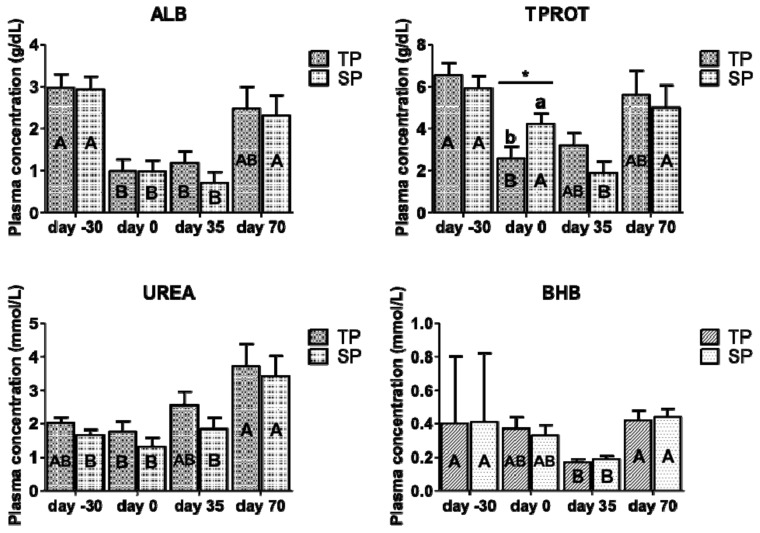
Metabolic response (ALB, TPROT, urea and BHB) in plasma of twin- and single bearing ewes according to time (LSM ± SEM). TP: twin-bearing ewes (n = 6); SP: single-bearing ewes (n = 7); ALB: albumin; TPROT: total protein; BHB: β-hidroxybutirate; day -30: 30 days pre-partum; day 0: birth, day 35: day 35 post-partum and day 70: day 70 post-partum; different small letters (a, b) denote significant differences between groups at *p* ≤ 0.05 (*); different capital letters (A, B) denote significant differences of each group according to time *p* ≤ 0.05 (*).

Figure 3 shows the metabolic response in plasma of twin- and single bearing ewes according to time, measured as ALB, TPROT, urea, and BHB. At birth (day 0), the group SP presented higher total protein proportions than group TP (*p* < 0.05). Except for cholesterol, each plasma indicator changed according to time. In both groups of ewes, the albumin and BHB concentrations increased at day −30 and day 70, but they were diminished at day 0 and day 35 (*p* < 0.05). In both groups of ewes, urea increased at day 70 (at weaning) (*p* < 0.05). No significant differences between groups or time were found for BHB in milk (*p* > 0.05).

**Figure 4:**
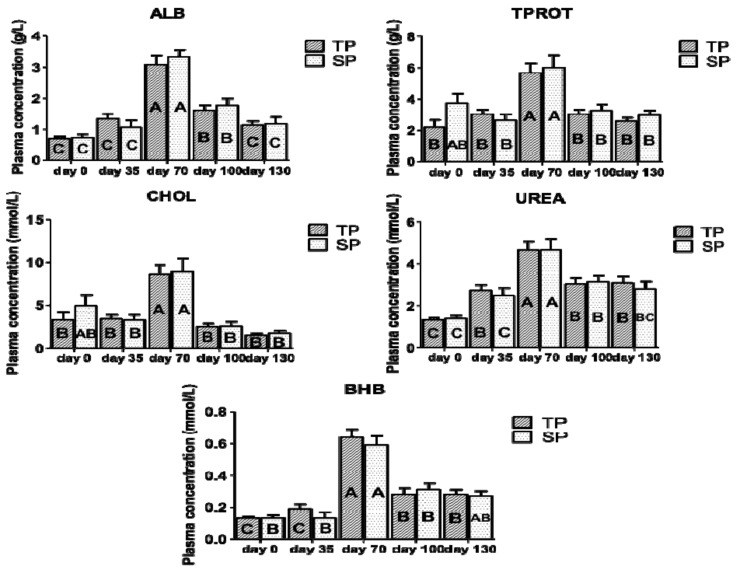
Metabolic response (ALB, TPROT, CHOL, urea and BHB) of lambs from twin- and single bearing ewes according to time (LSM ± SEM). TP: twin-bearing ewes (n = 6); SP: single-bearing ewes (n = 7); ALB: albumin; TPROT: total protein; CHOL: cholesterol; BHB: β-hidroxybutirate; day 0: birth, day 35: day 35 post-partum; day 70: day 70 post-partum (weaning); day 100: 30 days after weaning and day 130: 60 days after weaning; different capital letters (A, B, C) denote significant differences of each group according to time *p* ≤ 0.05.

Finally, Figure 4 shows the metabolic response (ALB, TPROT, CHOL, urea, and BHB) of lambs from twin- and single bearing ewes according to time (LSM ± SEM). Although no significant differences between treatments were found (*p* > 0.05), both of the groups overexpressed albumin, total protein, cholesterol, urea, and BHB at day 70 (at weaning). At day 0 (at birth), the group SP showed a trend to higher total protein proportions than group TP (*p* = 0.06).

The type of pregnancy had no effect on the lamb performance (*p* > 0.05; data included in Table S1) or on the transcriptional expression of lipogenic enzymes in the *Longissimus dorsi* muscle (*p* > 0.05), except for a trend to overexpress LPL in group SP when compared to group TP at day 0 (*p* = 0.08; data included in Table S2.

## 4. Discussion

It is a known fact that parity number affects the angiogenesis, turnover, and survival of mammary gland cells during lactation [10]. In the present study, it was possible to determine that the type of pregnancy affected VEGFR1 expression at day 0 (*p* < 0.05), noticing an overexpression in twin-bearing ewes at birth, a condition revealing the provision of angiogenic factors by the epithelial cells [39], which should follow the same developmental pattern than the aforementioned cells [10], although, in this case, the overexpression of VEGFR1 did not coincide with an overexpression of VEGF in the group of twin-bearing ewes at birth, which could be explained as a compensatory mechanism of the receptor in response of the under expression of VEGF or by a decrease of the VEGF protein synthesis. Therefore, it would be important to determine which factor regulates VEGF protein expression, which may have remained in the extracellular matrix, and it may have been degraded by proteases. On the other hand, VEFG increased its expression from day 0 to day 70 in the group of single-bearing ewes, following the same trend of its receptor VEGFR1, which was not significant.

In addition, CAIV was overexpressed in single-bearing ewes at day 35 of lactation (*p* < 0.05). The former is possibly explained by a slower mammary vascular network development in this group, in contrast to the group of twin-bearing ewes who finished this process before birth. The twin-bearing ewes overexpressed TBXAS1, a gene encoding for factors that regulate vascular tone and function [40], reaching a higher level at birth and day 35 than at the end of lactation, suggesting a higher expression of angiogenic factors, not only in the preparation of the mammary tissue for the next lactation, but also in the maintenance of lactation.

The occurrence of turnover/lactogenesis has been reported during late lactation [41,42], as observed in the present study, where the mRNA of IGF1 (but not its receptor IGF1R) was overexpressed in single-bearing ewes at day 35 post-partum, which was explained by the suckling induced IGF1 release in rats during lactation [43]. However, we noticed that the group of twin-bearing ewes did not overexpress IGF1 in spite of double suckling (*p* > 0.05). It is known that IGF1 is a primary mediator of the effects of growth hormone, being able to influence the growth of the mammary tissue and also tumorigenesis [44,45]. Therefore, future research should include the determination of the transcriptional expression of growth hormone and its receptor in the mammary gland tissue.

In our work, the mRNA of anti-apoptotic factor BCL2 [46] was overexpressed in twin-bearing ewes at birth and at day 35 of lactation and it showed a decreased expression from day 0 to day 70 (*p* < 0.05). A similar trend was observed in single-bearing ewes (*p* = 0.09), probably as a consequence of the end of lactation, although the results that were obtained for BAX, a pro-apoptotic factor [46], were not significant (*p* > 0.05). Concerning this point, Safayi et al. [10] reported that the anti-apoptotic factor BCL2 played a more important role than BAX in apoptosis regulation from birth to lactation. Thus, based on the results that were obtained by Gallardo et al. [30], we could infer that BCL2 was overexpressed in twin-bearing ewes, avoiding apoptosis and promoting a higher growth of the mammary tissue, which would be consistent with a higher milk production when compared to single-bearing ewes. However, in the present study, we could not be verified this point due to the NP-based diet quality.

It is necessary to point out that the present study included reports on other ruminants, since lactation is a process that is conserved across species and therefore suitable for comparison [47,48]. Thus, when considering the similar NP-based maternal diet, the type of pregnancy would explain the slight differences in mRNA expression of genes that are associated to angiogenesis and cell turnover/lactogenesis.

The type of pregnancy did not affect the plasma metabolic response in ewes, except for the higher total protein levels in single- than twin-bearing ewes at birth (*p* < 0.05; data included in Table S5), as explained by a lower energy expenditure of the single-bearing ewes by gestating a single lamb instead of two lambs. With the exception of BHB, all of the indicators were outside the reference limits that were reported for an adult sheep [49], a condition that is explained by the NP-based diet quality. The total protein proportion reveals whether the protein synthesis is adequate [14] and, although protein synthesis was below the reference limits for adult sheep [49], the proportions were higher in single- than twin-bearing ewes at birth (*p* < 0.05) on account of the higher energy demands by the group of twin-bearing ewes and, hence, an inadequate protein synthesis, especially at birth, being similar to the results that were obtained by Cárcamo et al. [50] working with twin and single-bearing ewes fed NP or red clover, who also reported that all groups exhibited a trend of higher protein levels when compared to the group of twin-bearing ewes fed NP at birth. As reported by Ríos et al. [51], BHB in milk was not affected by treatment (*p* > 0.05).

The type of pregnancy did not influence the plasma metabolic response in lambs (*p* > 0.05). All of the plasma indicators fell outside the reference limits reported in the literature, except for BHB [49]. Although twin and single-bearing ewes showed similar metabolites concentration in plasma, the higher values that were reported at day 70 post-partum could be explained in this period by the lambs suckling milk and also foraging, which decreased after weaning, when they had to get their own food to meet their nutrient requirements. The trend to higher total protein proportions in lambs from single- than twin-bearing ewes (*p* = 0.06; data included in Table S6) could be attributed to higher total protein levels in their mothers (*p* < 0.05).

In the present study, lamb performance was not affected by the type of pregnancy, which can be associated to the maternal diet supplemented with lupine and the BCS during pregnancy [2,52]. On the other hand, Cárcamo et al. [50] reported that both factors—the type of pregnancy and the diet—influenced lambs from single-bearing ewes fed red clover showing a trend to higher LBW at birth and day 30 post-partum, also showing a trend to higher body lengths than the lambs from the other treatments (*p* = 0.07). Furthermore, because the most relevant fetal growth takes place during the last 42 days of gestation, which is a period of increased energy demand, an important fraction of the ewe’s glucose is directed into the fetus-placental unit, producing a negative energy balance [53]. Thus, the above-mentioned detrimental condition did not allow lambs from single-bearing ewes to perform any better than lambs from twin-bearing ewes, making both groups exhibit a similar transcriptional expression of lipogenic enzymes in the *Longissimus dorsi* muscle (*p* > 0.05).

Therefore, when considering that all groups had a similar NP-based maternal diet, the type of pregnancy would explain the minor differences in mRNA expression that were encountered in angiogenesis and cell turnover/lactogenesis.

## 5. Conclusions

The results showed that twin-bearing ewes overexpressed VEGFR1, a gene related to angiogenesis at birth, and BCL2, a gene that is related to cell turnover/lactogenesis at birth and day 35 post-partum; however, single-bearing ewes overexpressed CAIV, a gene related to angiogenesis and IGF1, a gene related to cell turnover/lactogenesis, at day 35 post-partum. Similar metabolite concentrations in blood plasma (*p* > 0.05) were found between groups of ewes. The plasma metabolic response in lambs was similar between groups and it did not influence their performance, where a similar transcriptional expression of lipogenic enzymes in the *Longissimus dorsi* muscle was observed. Therefore, when considering the same maternal diet, the type of pregnancy could explain the slight differences in mRNA expression of genes that are associated to angiogenesis and cell turnover/lactogenesis in the mammary gland, although these differences not only did not affect the plasma metabolic response and milk secretion capacity in ewes, but they also had no influence on the performance, plasma metabolic response, and muscle transcriptional expression of lipogenic enzymes in their lambs.

## Figures and Tables

**Table 1:** Primer specifications.

Gene and Accession	Forward Primer Sequence	Amplicon
Angiogenesis	Reverse primer sequence	Length
*CAIV*	F: AGCGCTTTGCCATGGAGATACA	148
XM_012186664.1	R: AGGGGCTGGAAGTTCACATTCTTG	
*VEGF*	F: TGCTCTACCTTCACCATGCCAA	101
NM_001025110.1	R: GCGCTGGTAGACATCCATGAACTT	
*VEGFR1 (FTL1)*	F: AGGTGACCTGCTTCAAGCCAAT	106
XM_015098156.1	R: GAAGGCAGGTGTCGAGTACGTAAA	
*VEGFR2 (KDR)*	F: AAGACGCTGACTTGCTTTGGGA	150
NM_001278565.1	R: AAATGGGAAGAGCACGCAACCT	
*ANGPT1*	F: GCACCCTCATGCATTCTTGTCA	140
XM_004011787.3	R: ACCCTTTCCTCTACCCTATCTGCT	
*ANGPT2*	F: GAGACCTGCTCCCAAAGCAGTAAA	145
XM_004021671.3	R: TCACTGAGTGATGCGGGTTCAA	
*MKI67*	F: TGCAGACTTTGGCACAAACGAC	143
XM_015103501.1	R: AGTTTTAGCAGGACGCCTGGAA	
*TBXAS1*	F: CATCTTCCTCATTGCTGGCTACGA	143
XM_012177234.2	R: AGTACTCAGGGGCTGGATGTTTCT	
Cell turnover		
*LALBA*	F: TGCCACCCAGGCTGAACAATTA	106
NM_001009797.1	R: AAATGCGGTACAGACCCATTCAGG	
*BAX*	F: CTAAGACCTGGTGTAGCCAAGCAA	103
XM_015100639.1	R: TCGAACCCATGTTCCCTGCATT	
*BCL2*	F: ATGCGGCCCCTGTTTGATTTCT	112
XM_012103831.2	R: GTGGACTTCACTTATGGCCCAGAT	
*CCND1*	F: ACGACTTCATCGAGCACTTCCTCT	127
XM_015102997.1	R: GGTGGGTTGGAAATGAACTTCACG	
*IGF1*	F: CCAGACTTTGCACTTCAGAAGC	106
XM_012159642.2	R: GATGTGACTGGCATCTTCACCT	
*IGF1R*	F: CGAGATCCTGTACATTCGCACCAA	100
XM_012098367.2	R: GTTCCACTTCACGATCAGCTGAGA	
*IGFBP1*	F: CAGCGATGAGGCCACAGATACAAA	117
NM_001145177.1	R: CTGGACTCGGTCATCAAGTGGAAA	
*IGFBP3*	F: AGGTTGACTACGAGTCTCAGAGCA	122
NM_001159276.1	R: CAGGAACTTGAGGTCGTTCAGTGT	
*IGFBP5*	F: TGCGTGGACAAGTATGGGATGAAG	103
NM_001129733.1	R: AGGGGACGCATCACTCAACATT	
*LPT*	F: ATCCCACTCACCAGCATGCAAA	145
XM_004008038.3	R: CTACCAAGTGCAAGCACAGTTAGC	
*LPTR*	F: TTGGATGGCCTAGGAATCTGGAGT	105
NM_001009763.1	R: GTTAGACCCAACCGCTGTCAGAAT	
*LTF*	F: GGTTATTCTGGTGCCTTCAAGTGC	119
NM_001024862.1	R: AGAAGCTCATACTGGTCCCTGTCA	
*CYP19A*	F: AACACGTCCACATAGCCCAAGT	80
NM_001123000.1	R: ACCATCTGTGCTGATTCCATCACC	
*TGFB1*	F: GCACGTGGAGCTGTACCAGAAATA	116
NM_001009400.1	R: GCACAACTCCAGTGACGTCAAA	
*TGFB1R1*	F: CCAAGGAAAACCAGCCATAGCTCA	118
XM_012120354.2	R: TGTGGCCGAATCATGCCTTACT	
*TGFB1R2*	F: CCTTACAAAGCATGTGGGCTTGAC	132
*XM_012099307.2*	R: CCTGCACTGTAGGCGGATTCTTTA	
*ACTIN*	F: TGAAGTGTGACGTGGACATCCGTA	108
NM_001009784.1	R: AGGTGATCTCCTTCTGCATCCTGT	

CAIV: Carbonic Anhydrase IV; VEGF: vascular endothelial growth factor A; VEGFR1 (FTL1): fms-related tyrosine kinase 1; VEGFR2 (KDR): kinase insert domain receptor; ANGPT1: angiopoietin 1; ANGPT2: angiopoietin 2; MKI67: marker of proliferation Ki-67; TBXAS1: thromboxane A synthase 1; LALBA: lactalbumin alpha; BAX: BCL2-associated X protein; BCL2: B-cell CLL/lymphoma 2; CCND1: cyclin D1; IGF1: insulin like growth factor 1; IGF1R; IGFBP1: insulin like growth factor binding protein 1; IGFBP3: insulin like growth factor binding protein 3; IGFBP5: insulin like growth factor binding protein 5; LPT: leptin; LPTR: leptin receptor; LTF: lactotransferrin; TGFB1: transforming growth factor beta 1; TGFB1R1: transforming growth factor, beta receptor 1; TGFB1R2: transforming growth factor, beta receptor 2; actin: β-actin.

**Table 2:** Chemical analysis of the pastures during three consecutive measurements.

Pastures	DM ^6^	TA ^7^	CF ^8^	CP ^9^	ME ^10^	NDF ^11^
NPH ^1^	87.68 ± 0.98	5.78 ± 0.30	1.75 ± 0.09	6.83 ± 0.10	1.90 ± 0.13	65.89 ± 2.20
NP (at):						
Birth ^2^	20.98 ^d^ ±0.03	8.78 ^c^ ± 0.05	2.56 ^c^ ± 0.01	27.91 ^a^ ± 9.31	3.07 ^a^ ± 0.02	46.88 ^c^ ± 0.01
35d a B. ^3^	20.28 ^e^ ± 0.03	8.66 ^c^ ± 0.09	2.52 ^c^ ± 0.02	22.23 ^b^ ± 0.06	3.07 ^a^ ± 0.02	45.71 ^e^ ± 0.02
70d (weaning)	21.35 ^c^ ± 0.05	7.88 ^d^ ± 0.01	2.49 ^d^ ± 0.04	17.84 ^d^ ± 0.03	2.56 ^c^ ± 0.01	59.17 ^a^ ± 0.02
30d a. W. ^4^	28.10 ^b^± 0.03	9.50 ^b^ ± 0.02	3.06 ^b^ ± 0.01	21.57 ^c^ ± 0.02	2.77 ^b^ ± 0.02	46.46 ^d^ ± 0.03
60d a. W. ^5^	28.25 ^a^ ± 0.02	9.95 ^a^ ± 0.01	4.26 ^a^ ± 0.01	15.48 ^e^ ± 0.04	2.55 ^c^ ± 0.01	55.17 ^b^ ± 0.02
*p* value	<0.0001	<0.0001	<0.0001	<0.0001	<0.0001	<0.0001

^1^ NPH: naturalized pasture hay; ^2^ NP: naturalized pasture at birth; ^3^ 35d a. B.: NP 35days after birth; ^4^ 30d a.W.; NP 30 days after weaning; ^5^ 60d a. W.: NP 60 after weaning; ^6^ DM: dry matter (%); ^7^ TA: total ashes (%);^8^ CF: crude fat (%);^9^ CP: crude protein (%); ^10^ ME: metabolizable energy (kg DM^−1^); ^11^ NDF: neutral detergent fiber (%) (n = 3); Different small letters (a–e) denote significant differences between groups at *p* ≤ 0.05.

## Supplementary Materials

The following are available online at https://www.mdpi.com/2076-2615/10/12/2290/s1. Table S1. Effect of the type of pregnancy (twin or single lamb) on lamb performance including LBW (Kg) and ADG at 35d, at weaning and also at 30 and 60 days after weaning (LSM ± SEM) ^1,2,3,4,5^, Table S2. Final effect of the type of gestation (twin or single lamb) on the transcriptional expression of ACC, FAS, SCD1 and SREBP1c, LPL and PPARγ in *Longissimus dorsi* muscle in lambs from weaning (70 days) to 30 and 60 days after weaning (LSM ± SEM), Table S3. Effect of the type of pregnancy on the transcriptional expression of genes associated to angiogenesis inside the mammary gland (LSM ± SEM), Table S4. Effect of the type of pregnancy on the transcriptional expression of genes associated to cell turnover/lactogenesis inside the mammary gland (LSM ± SEM), Table S5. Effect of the type of pregnancy and the measuring time related on plasma metabolic response in ewes (LSM ± SEM) ^1,2,3,4,5^, Table S6. Effect of the type of pregnancy and the measuring time related on plasma metabolic response in lambs (LSM ± SEM) (TP; n = 6); (SP; n = 7) ^1,2,3,4^.
