# Supplementary material for: Effect of Type of Pregnancy on Transcriptional and Plasma Metabolic Response in Sheep and Its Further Effect on Progeny Lambs
Source: Animals (Basel). 2020 Dec 3;10(12):2290. doi: 10.3390/ani10122290 (PMC7761827; doi:10.3390/ani10122290)
Supplement: Supplementary file 1 [file animals-10-02290-s001.pdf]

Supplementary Materials:

Table S1. Effect of the type of pregnancy (twin or single lamb) on lamb performance including LBW (Kg) and ADG at 35days, at weaning and also at 30 and 60 days after weaning (LSM ± SEM) [1,2,3,4, 5.]

Groups	LBW[1]	ADG 35d[2]	ADG 70d W.[3]	ADG 30d a.W.[4]	ADG60d a.W.[5]
Birth					
TP[6]	7.18 ± 0.52				
SP[7]	7.41 ± 0.69				
P value	0.79				
Day 35					
TP	21.00 ± 1.11	0.39 ± 0.02			
SP	21.31 ± 1.46	0.40 ± 0.03			
P value	0.86	0.95			
Day 70 (Weaning)					
TP	24.08 ± 1.38		0.24 ± 0.01		
SP	26.94 ± 1.81		0.27 ± 0.02		
p value	0.22		0.15		
30 days a.W					
TP	28.17 ± 1.90			0.21 ± 0.01	
SP	27.54 ± 2.44			0.20 ± 0.02	
p value	0.80			0.76	
60 days a.W.					
TP	25.72 ± 1.26				0.14 ± 0.01
SP	26.57 ± 1.65				0.15 ± 0.01
p value	0.68				0.58

[1]LBW: Live Body weight (kg); [2]ADG: Average daily gain at 35d; [3]ADG at 70 d (ADG at 70 days, weaning); [4]ADG 30days a.W.: ADG at 30 days after weaning; [5]ADG 60 days a.W.: ADG at 60 days after weaning; [6]TP: twin-bearing ewes fed NP (n = 6); [7]SP: single-bearing ewes fed NP (n = 7); Different small letters (a,b) denote significant differences between groups at $p \leq 0.05$.

Table S2. Final effect of the type of gestation (twin or single lamb) on the transcriptional expression of ACC, FAS, SCD1 and SREBP1c, LPL and PPARγ in *Longissimus dorsi* muscle in lambs from weaning (70 d) to 30 and 60 days after weaning (LSM ± SEM).

PERIOD	ACC[1]	FAS[2]	SCD1[3]	SREBP1c[4]	LPL[5]	PPAR[6]γ
Day 70 (Weaning)						
TP[7]	1.88 ± 0.57	2.15 ± 0.75	0.98 ± 0.30	1.30 ± 0.32	1.02 ± 0.66	1.44 ± 0.51
SP[8]	2.25 ± 0.70	1.41 ± 0.87	1.82 ± 0.37	1.20 ± 0.40	2.94 ± 0.81	1.91 ± 0.63
P groups[9]	0.68	0.51	0.10	0.84	0.08	0.56
30 days after weaning						
TP	1.57 ± 0.52	1.49 ± 0.44	1.35 ± 0.31	1.07 ± 0.28	1.61 ± 0.46	1.57 ± 0.40
SP	1.92 ± 0.63	1.25 ± 0.54	1.20 ± 0.38	1.46 ± 0.34	1.12 ± 0.56	1.05 ± 0.48
P groups	0.67	0.74	0.76	0.39	0.50	0.42
60 days after weaning						
TP	1.56 ± 0.47	1.81 ± 0.47	1.56 ± 0.40	1.74 ± 0.49	1.37 ± 0.41	1.54 ± 0.41
SP	1.57 ± 0.58	1.11 ± 0.57	1.34 ± 0.49	1.15 ± 0.60	1.55 ± 0.50	1.24 ± 0.50
P groups	0.99	0.35	0.72	0.45	0.78	0.65
P times						
TP	0.89	0.77	0.52	0.50	0.66	0.98
SP	0.74	0.87	0.49	0.83	0.21	0.29

[1]ACC: acetyl-CoA carboxylase; [2]FAS: fatty acid synthase; [3]SCD1: stearoyl CoA desaturase 1; [4]SREBP1c: sterol regulatory element binding transcription factor 1c; [5]LPL: Lipoprotein lipase; [6]PPARγ: Peroxisome proliferator-activated receptor gamma; [7]TP: twin-bearing ewes fed NP (n = 6); [8]SP: single-bearing ewes fed NP (n = 6); [9]Different small letters (a,b) denote significant differences between groups at $p \leq 0.05$.

Table S3. Effect of the type of pregnancy on the transcriptional expression of genes associated to angiogenesis inside the mammary gland (LSM ± SEM).

Groups	CAIV	VEGF	VEGFR1	VEGFR2	ANGPT1	ANGPT2	MK167	TBXAS1
Day 0 (Birth)								
TP[1]	1.83 ± 0.31	1.39 ± 0.22	1.70a ± 0.24	1.58 ± 0.28	1.12 ± 0.14	1.57 ± 0.28	1.37 ± 0.76	1.43 ± 0.21AB
SP[2]	1.06 ± 0.31B	1.08 ± 0.22B	0.84b ± 0.24	0.94 ± 0.28	0.98 ± 0.14	0.83 ± 0.28	1.67 ± 0.76	0.86 ± 0.21
P groups[3]	0.11	0.33	0.03	0.14	0.51	0.09	0.78	0.09
Day 35								
TP	0.74b ± 0.61	0.96 ± 0.12	1.05 ± 0.39	1.12 ± 0.40	0.75 ± 0.20	1.55 ± 0.35	1.64 ± 0.43	1.86 ± 0.38A
SP	3.23a±0.61 A	1.13 ± 0.12B	1.68 ± 0.39	1.38 ± 0.40	1.37 ± 0.20	0.98 ± 0.35	1.34 ± 0.43	1.01 ± 0.38
P groups	0.01	0.34	0.28	0.65	0.06	0.28	0.62	0.15
Day 70								
TP	1.34 ± 0.40	0.96 ± 0.41	1.27 ± 0.54	1.68 ± 0.33	1.13 ± 0.49	1.16 ± 0.48	1.64 ± 1.00	0.83 ± 0.35B
SP	1.61 ± 0.40B	2.26 ± 0.41A	1.70 ± 0.54	0.98 ± 0.33	1.66 ± 0.49	1.54 ± 0.48	2.11 ± 1.00	1.61 ± 0.35
P groups	0.65	0.06	0.59	0.17	0.47	0.58	0.74	0.14
P times[4]								
TP	0.08	0.45	0.44	0.52	0.61	0.67	0.96	0.006
SP	0.04	0.01	0.35	0.56	0.36	0.43	0.76	0.43

[1]TP: ewes gestating twins on NP (n = 6); [2]SP: ewes single lamb on NP (n = 7); [3]Different small letters (a,b) denote significant differences between groups (TP and SP) within each measuring time at $p \leq 0.05$; [4]Different capital letters (A, B) denote significant differences of each group according to time (day 0 (birth), day 35 post-partum and day 70 post-partum) at $p \leq 0.05$.

Table S4. Effect of the type of pregnancy on the transcriptional expression of genes associated to cell turnover/ lactogenesis inside the mammary gland (LSM ± SEM).

Groups	LALBA	BAX	BCL2	CCND1	IGF1	IGF1R	IGFBP3	IGFBP5	LPT	LPTR	LTF	TGFB1	TGFB1R1
Day 0 (Birth)													
TP[3]	1.23±0.21	1.52 ± 0.34	1.40a ± 0.13A	1.12 ± 0.18	1.39 ± 0.38	1.28 ± 0.24	1.59 ± 0.27	1.01 ± 0.21	1.21 ± 0.65	1.28 ± 0.17	2.20 ± 0.53	1.04 ± 0.22	1.37 ± 0.23
SP[1]	0.92 ±021	0.94 ± 0.34	0.76b ± 0.13	1.05 ± 0.18	0.96± 0.38B	0.98 ± 0.24	0.78 ± 0.27	1.18 ± 0.21	1.84 ± 0.65	0.92 ± 0.17	0.92 ± 0.53	1.19 ± 0.22	0.89 ± 0.23
P Groups[3]	0.31	0.26	0.009	0.78	0.45	0.40	0.06	0.58	0.51	0.17	0.12	0.65	0.17
Day 35													
TP	2.22±0.35	2.12 ± 0.65	1.41a ± 0.16A	0.90 ± 0.42	0.77b ± 0.65	0.91 ± 0.54	1.65 ± 0.45	1.04 ± 0.38	3.41 ± 1.02	0.98 ± 0.28	2.34 ± 1.14	0.94 ± 0.58	0.92 ± 0.31
SP	1.51±0.35	1.13 ± 0.65	0.83b ± 0.16	1.69 ± 0.42	3.71a ± 0.65A	1.63 ± 0.54	0.92 ± 0.45	1.58 ± 0.38	1.95 ± 1.02	1.27 ± 0.28	2.46 ± 1.14	2.17 ± 0.58	1.46 ± 0.31
P Groups	0.18	0.31	0.03	0.22	0.01	0.36	0.28	0.34	0.33	0.47	0.94	0.16	0.25
Day 70													
TP	1.39±0.50	2.18 ± 0.78	0.88 ± 0.22B	1.77 ± 0.70	2.44 ± 0.55	1.31 ± 0.39	1.13 ± 0.34	0.94 ± 0.47	2.27 ± 0.86	0.91 ± 0.43	2.08 ± 0.71	1.01 ± 0.54	0.93 ± 0.28
SP	1.97±0.50	1.72 ± 0.78	1.38 ± 0.22	2.07 ± 0.70	1.19± 0.55B	1.15 ± 0.39	1.26 ± 0.34	1.76 ± 0.47	1.57 ± 0.86	1.62 ± 0.43	1.85 ± 0.71	1.84 ± 0.54	1.41 ± 0.28
PGroups	0.43	0.68	0.14	0.76	0.14	0.77	0.79	0.25	0.58	0.27	0.82	0.31	0.26
Ptimes[4]													
TP	0.10	0.74	0.03	0.10	0.12	0.64	0.69	0.97	0.17	0.30	0.96	0.97	0.38
SP	0.24	0.59	0.09	0.52	0.006	0.60	0.26	0.60	0.95	0.49	0.53	0.49	0.35

For footnotes, see Table 3.

Table S5. Effect of the type of pregnancy and the measuring time related on plasma metabolic response in ewes (LSM ± SEM) [1,2,3,4,5,.]

Groups	**ALB[1]** (g / dL)	**TPROT[2]** (g / dL)	**CHOL[3]** (mmol / L)	**UREA** (mmol / L)	**BHB[4]** (mmol / L)	**BHB-M[5]** (mmol / L)
Day -30						
TP[6]	2.97 ± 0.32A	6.53 ± 0.60A	5.38 ± 0.63	2.01 ± 0.17AB	0.40 ± 0.06A	
SP[7]	2.93 ± 0.30A	5.93 ± 0.56A	4.75 ± 0.59	1.67 ± 0.15B	0.41 ± 0.06A	
P groups[3]	0.93	0.47	0.48	0.17	0.86	
Day 0 (birth)						
TP	0.98 ± 0.29B	2.58b ± 0.55B	2.95 ± 1.17	1.77 ± 0.30B	0.37 ± 0.07AB	0.26 ± 0.02
SP	0.97 ± 0.27B	4.21a ± 0.51A	5.01 ± 1.09	1.30 ± 0.28B	0.33 ± 0.06AB	0.24 ± 0.02
P groups	0.97	0.04	0.22	0.43	0.69	0.33
Day 35						
TP	1.18 ± 0.27B	3.18 ± 0.62 AB	2.86 ± 0.67	2.55 ± 0.41AB	0.17 ± 0.02B	0.18 ± 0.06
SP	0.71 ± 0.25B	1.87 ± 0.57B	1.51 ± 0.63	1.84 ± 0.38B	0.19 ± 0.02B	0.20 ± 0.03
P groups	0.22	0.14	0.16	0.22	0.45	0.09
Day 70						
TP	2.48 ± 0.52AB	5.60 ± 1.15AB	4.98 ± 1.22	3.71 ± 0.66A	0.42 ± 0.06A	0.23 ± 0.09
SP	2.31 ± 0.48A	4.99 ± 1.07A	4.84 ± 1.13	3.41 ± 0.61A	0.44 ± 0.05A	0.20 ± 0.04
P groups	0.81	0.70	0.93	0.74	0.73	0.63
P times[4]						
TP	0.004	0.01	0.10	0.008	0.01	0.63
SP	0.0001	0.001	0.07	0.0005	0.005	0.36

[1]ALB: albumin; [2]TPROT: total protein; [3]CHOL: cholesterol; [4]BHB: β-hidroxybutirate; [5]BHB-M: BHB in milk; [6]TP: twin-bearing ewes fed NP (n = 6); [7]SP: single-bearing ewes fed NP (n = 7); [3]Different small letters (a,b) denote significant differences between groups (TP and SP) at $P \leq 0.05$; [4]Different capital letters (A,B) denote significant differences of each group according to time (day -30 pre-partum; day 0 (birth), day 35 post-partum and day 70 post-partum) at $p \leq 0.05$.

Table S6. Effect of the type of pregnancy and the measuring time related on plasma metabolic response in lambs (LSM ± SEM) (TP; n = 6); (SP; n = 7) [1,2,3,4].

**Groups**	**ALB[1]** (g / dL)	**TPROT[2]** (g / dL)	**CHOL[3]** (mmol / L)	**UREA** (mmol / L)	**BHB[4]** (mmol / L)
Day 0 (birth)					
TP[5]	0.70 ± 0.08C	2.19 ± 0.48B	3.25 ± 0.95B	1.30 ± 0.11C	0.13 ± 0.01C
SP[6]	0.73 ± 0.11C	3.73 ± 0.62AB	4.92 ± 1.24AB	1.35 ± 0.14C	0.13 ± 0.02B
P groups	0.83	0.06	0.30	0.80	0.87
Day 35					
TP	1.33 ± 0.17BC	3.03 ± 0.31B	3.38 ± 0.48B	2.70 ± 0.26B	0.19 ± 0.03BC
SP	1.06 ± 0.23BC	2.63 ± 0.41B	3.22 ± 0.63B	2.47 ± 0.35BC	0.13 ± 0.04B
P groups	0.35	0.44	0.84	0.61	0.26
Day 70					
TP	3.08 ± 0.30A	5.67 ± 0.61A	8.54 ± 1.17A	4.63 ± 0.40A	0.64 ± 0.05A
SP	3.33 ± 0.22A	6.00 ± 0.79A	8.94 ± 1.53A	4.64 ± 0.52A	0.59 ± 0.06A
P groups	0.62	0.74	0.83	0.98	0.46
Day 100					
TP	1.61 ± 0.17B	3.03 ± 0.31B	2.45 ± 0.40B	3.01 ± 0.29B	0.28 ± 0.043B
SP	1.78 ± 0.22B	3.26 ± 0.41B	2.51 ± 0.53B	3.12 ± 0.31B	0.31 ± 0.04B
P groups	0.70	0.66	0.92	0.93	0.96
Day 130					
TP	1.13 ± 0.13BC	2.58 ± 0.24B	1.47 ± 0.21B	3.05 ± 0.31B	0.28 ± 0.03B
SP	1.17 ± 0.23BC	2.96 ± 0.31B	1.71 ± 0.28B	2.77 ± 0.36BC	0.27 ± 0.03AB
P groups	0.40	0.35	0.51	0.89	0.70
P times[7]					
TP	<0.0001	<0.0001	<0.0001	<0.0001	<0.0001
SP	<0.0001	0.0023	0.0007	<0.0001	<0.0001

[1]ALB: albumin; [2]TPROT: total protein; [3]CHOL: cholesterol; [4]BHB: β-hidroxybutirate; [5]TP: twin-bearing ewes fed NP (n = 6); [6]SP: single-bearing ewes fed NP (n = 7); [7]Different capital letters (A, B) denote significant differences of each group according to time (day 0 (birth), day 35 post-partum and day 70 post-partum (weaning), day 30 after weaning and day 60 after weaning, at $p \leq 0.05$.
